# Multi‐family therapy for eating disorders across the lifespan: A systematic review and meta‐analysis

**DOI:** 10.1002/erv.2919

**Published:** 2022-06-21

**Authors:** Jennifer Zinser, Nicola O’Donnell, Lucy Hale, Christina J. Jones

**Affiliations:** ^1^ School of Psychology University of Surrey Guildford UK

**Keywords:** eating disorders, meta‐analysis, multi‐family therapy, parents, systematic review

## Abstract

Eating disorders (EDs) have an estimated prevalence rate of 1%–5% across Europe. Effective adjunct interventions are needed to support the 20%–40% of families whose recovery requires additional support to first line approaches. This systematic review and meta‐analysis aimed to establish whether multi‐family therapy (MFT) improves the physical and psychological health of patients and family members. Searches were conducted in PsycINFO, MEDLINE, PubMed, EMBASE, CINAHL, and the Cochrane Library in March 2021. 15 studies (850 patients) met the inclusion criteria. Meta‐analysis demonstrated MFT resulted in significant benefits in weight gain, ED symptoms, patients' and parents' depression symptoms, and parents' negative experiences of caregiving. However, significant improvements were only evident when comparisons were drawn before and after the intervention; these dissipated when MFT was compared to another intervention. There was no evidence MFT improves family functioning, positive aspects of caregiving, nor patient and parental anxiety. Intervention completion rates ranged from 86% to 100% indicating a high level of acceptability. Studies varied with regard to intervention length and structure, follow‐up period, and outcome measures utilised; most were rated as moderate or weak in methodological quality. More rigorous and large scale randomised controlled trials are needed to fully assess the effectiveness of MFT.

## Key points

- Multi‐family therapy for eating disorders results in significant benefits in weight gain, ED symptoms, patients' and parents' depression symptoms, and parents' negative experiences of caregiving.
- However, significant improvements dissipated when MFT was compared to another intervention.
- Adherence to multi‐family therapy is very good, indicating a high level of acceptability.
- More rigorous and large scale randomised controlled trials are needed to fully assess the effectiveness of MFT.

**Abbreviations**
%EBW: percentage expected body weight
%IBW: percentage ideal body weight
AN: anorexia nervosa
ARFID: avoidant restrictive food intake disorder
BDI: Beck Depression Inventory
BMI: body mass index
BN: bulimia nervosa
CDI: children's depression inventory
CGAS: children's global assessment scale
DCCFS: devaluation of consumers and consumer families questionnaire
DERS: difficulties in emotion regulation scale
ECI: experience of caregiving inventory
ED: eating disorder
EDE‐Q: eating disorder examination questionnaire
EDI: Eating Disorder Inventory
EDNOS: eating disorder not otherwise specified
EDSIS: eating disorder impact scale
FAD: family assessment device
FQ: family questionnaire
GHQ‐12: general health questionnaire 12
HADS: hospital anxiety and depression scale
IIP: inventory of interpersonal problems
LEE: perceived level of expressed emotion
OQ‐45: outcome questionnaire 45
OSFED: other specified feeding or eating disorder
RCADS: revised children's anxiety and depression scale
RSES: Rosenberg's self‐esteem scale
SASB‐Intrex: structural analysis of social behaviour
SEED: short evaluation of eating disorders
SOS‐10: Schwartz outcome scale 10
SPS: social provisions scale
STAI: Spielberger state‐trait anxiety inventory
TAU: treatment as usual

## 1 INTRODUCTION

### 1.1 Rationale

Eating disorders (EDs) are characterised by excessive pre‐occupations with shape and weight and are reported to affect between 1% and 4% of women and 0.3%–0.7% of men in Europe (Keski‐Rahkonen & Mustelin, 2016). Anorexia nervosa (AN) is noted to have the highest mortality rate of psychiatric illnesses, approximately 5% across the lifetime (Arcelus et al., 2011; Steinhausen et al., 2008; Sullivan, 1995). Many people who receive outpatient support as an adolescent do not fully recover: approximately 20%–40% either relapse or require further treatment (Berends et al., 2018; Steinhausen, 2002; Zipfel et al., 2000). The chronic nature of the illness combined with medical and psychiatric comorbidities leads to high levels of costly health services utilisation (Green & Griffiths, 2014; Tseng et al., 2021), highlighting the need for effective early identification and intervention.

Initial theories posited the family environment contributed to EDs, resulting in treatments focussing on weight restoration in an inpatient setting, with the child removed from the family unit (Treasure & Schmidt, 2013). An ideological shift occurred in how the role of the family was understood in relation to EDs in the 1960s (Le Grange & Eisler, 2009) marked by the proposal of alternative theories, notably Minuchin et al.’s (1978) psychosomatic family model (Minuchin et al., 1978). The model sparked the development of treatments where family need to take an active role in treatment to facilitate changes in patterns of family functioning.

In more recent years, ED‐based family therapy has become manualised and includes similar forms of evidence‐based interventions for example, Family‐Based Treatment (FBT; Lock & Le Grange, 2013), the Maudsley Model of Family Therapy (FT‐AN; Eisler et al., 2016a) and Systemic Family Therapy (SyFT; Agras et al., 2014). The first‐line recommended treatment for children and adolescents with an ED is family therapy (National Institute for Health and Care Excellence, 2017). In the community and in several randomised controlled trials (RCTs), FBT has been show as an effective form of early invention (Lock et al., 2019). The manualised FBT and FT‐AN approaches overlap in their adoption of a phased approach and focus on utilising family strengths, enabling them to support their child's recovery. Initial phases focus on weight restoration with parents taking control of their child's eating, followed by the child gradually regaining autonomy of this, and lastly addressing individual and family concerns outside of the ED (Rienecke, 2017).

Multi‐family therapy (MFT) is an umbrella term encapsulating a variety of approaches bringing together several families affected by the same concern (Gelin et al., 2018). MFT is grounded in group therapy, based on the idea that families learn from one another in supporting their loved one (Asen & Schuff, 2006). Broadly, MFT in the ED context is developed from the described single‐family approach, also drawing upon aspects of structural, systemic, narrative and psychodrama modalities (Dare & Eisler, 2000).

A number of manualised MFT approaches for ED patients and their families have now been developed, typically offered as an adjunct treatment. These include those developed for children and adolescents with AN at the Maudsley Hospital (MFT‐AN; Simic et al., 2021), multiple family therapy adolescents with disordered eating in Dresden, Germany (Scholz & Asen, 2001), multi‐family therapy groups for adults (MFTG; Tantillo et al., 2020), and MFT for bulimia nervosa (BN; Stewart et al., 2015) which also incorporates elements of cognitive behavioural therapy and dialectical behavioural therapy.

MFT typically involves three to eight families attending a 3‐ or 4‐day intensive programme followed by monthly sessions over 6–9 months. Families may have additional single‐family sessions in between these as needed. Sessions last for 1 day, including a family meal plus snacks, and are facilitated by two to four clinicians (Jewell et al., 2016). However, variability does exist in the structure and some approaches adopt a more intensive schedule of three (Whitney et al., 2012) or five (Wierenga et al., 2018) days without follow‐ups, or shorter but more frequent sessions (Dimitropoulos et al., 2015; Tantillo et al., 2019). Despite this variability, all models aim to improve outcomes by reducing isolation and supporting families to rediscover their strengths and gain confidence tackling difficulties (Simic & Eisler, 2015).

Preliminary evidence has suggested MFT may be an encouraging alternative to FBT in terms of weight gain and reducing ED symptoms and carer burden (Murray & Le Grange, 2014). As an intensive treatment, it utilises a high level of resources, however in Germany, MFT was found to cost 25% of the average cost of inpatient ED care (Gelin et al., 2018; Scholz et al., 2005). MFT is currently being implemented into clinical services across the world, in line with NICE guidelines which outline that family therapy may be offered together with other families (National Institute for Health and Care Excellence, 2017). However, despite this, the approach remains relatively understudied. Family approaches are recommended for treating AN in children and adolescents, however individual approaches are prioritised for adults, with some flexibility to involve carers as appropriate (National Institute for Health and Care Excellence, 2017). MFT has been applied to both adult and adolescent patient groups, however there is currently a lack of clarity surrounding the relative efficacy of the approach amongst these different populations. Since this review was prospectively registered, a systematic scoping review into MFT for EDs has been published which included both quantitative and qualitative studies (Baudinet et al., 2021) though no meta‐analyses were conducted. The novel data generated by this meta‐analysis looking at the key outcomes of MFT aims to further contribute to our understanding of the treatment's efficacy, including differences between adult and adolescent samples. In addition, further assessment of treatment response and retention rates and questionnaire completion hopes to shed light on the intervention's acceptability.

### 1.2 Objectives

This systemic review and meta‐analysis had three objectives:

- To determine the impact of MFT on patient's weight and ED symptomatology, as well as on patient and carer psychological well‐being. This includes subgroup analyses of adult compared to adolescent samples where the data allows for this.
- To determine the impact of MFT on family functioning and caregiver burden.
- To determine the acceptability of MFT as an intervention for EDs, and the acceptability of outcome measures utilised.

## 2 METHODS

This review followed the Preferred Reporting Items for Systematic Reviews and Meta‐analyses guidelines (PRISMA; Moher et al., 2009). The protocol was prospectively published on Prospero (CRD42021229462).

### 2.1 Eligibility criteria

Studies were eligible for inclusion if they (1) included participants with a diagnosis of an ED (this includes AN, BN, other specified feeding or eating disorder [OSFED], eating disorder not otherwise specified [EDNOS], binge eating disorder [BED], and/or avoidant restrictive food intake disorder [ARFID]), (2) at least one participant in the study received MFT for an eating disorder, (3) therapy involved at least one family member or person of support in addition to the person with the diagnosis, and (4) measured intervention effectiveness using quantitative outcomes. Definitions of MFT can vary, and studies were excluded if the intervention consisted of psychoeducation only or was only provided to family members or carers. Studies were eligible for inclusion regardless of participant age, structures of family or support system, whether they had a comparison group, and what this comparator consisted of. Studies were excluded if they solely described the intervention or solely collected qualitative data. Studies that used subsets of data published in full elsewhere were not included to prevent duplication of data.

### 2.2 Search strategy

Keyword searches were carried out in PsycINFO, MEDLINE, PubMed, EMBASE, CINAHL, and the Cochrane Library (Appendix 1) from database inception to the search date (March 2021). Publication type was limited to peer reviewed journals: time and resource constraints prevented the inclusion of grey literature. Citations were screened by one reviewer (JZ) and all were independently checked by a second reviewer (NOD). Discrepancies (*n* = 6) were discussed and both reviewers confirmed the eligibility of the identified studies.

### 2.3 Data extraction

Relevant data were extracted into a summary table to enable comparisons of study characteristics (Table 1). The table was compiled by one reviewer (JZ) and checked for accuracy by another reviewer (NOD). In circumstances where studies were eligible for inclusion but not all relevant information was included in the article, authors were contacted.

**TABLE 1:** Characteristics of included studies

Author, date, country, study design	Participants: patients	Participants: families	Intervention and comparator	Comparator	Follow‐up period	Outcome measures (patient‐completed unless specified)	Intervention refusal/drop out and outcome measure completion	Main findings
Dennhag et al. (2021), Sweden, Case series	24 adolescents	23 mothers and 22 fathers participated. Mean maternal age = 44, mean paternal age = 47. 79% of parents had a university degree. 100% of fathers and 60% of mothers worked full time.	Multi‐family therapy (MFT).	No comparison group.	At baseline and then post‐intervention at 1 year.	BMI, %EBW, eating disorder symptomatology (EDE‐Q), psychological well‐being (CGAS), familial burden (EDSIS, family).	Days of participation in the MFT by any of the parents ranged from 8 to 10 days. Overall dropouts not reported.	Patients significantly improved in self‐rated eating disorder symptoms, BMI, and global function pre‐ to post‐adjunct MFT. Parents reported a significant reduction in caregiver burden pre‐ to post‐MFT and also reported that their feelings of guilt decreased. No significant difference between the mother and father groups were found. Major associations were found between a decrease in parental burden of social isolation and adolescent recovery in BMI and daily function during treatment. For both mothers and fathers, the decrease in social isolation was strongly associated with physical treatment outcome.
	Diagnoses: AN (38%), EDNOS (62%)		Total contact hours: Approx. 74 (10.5 days)				No incomplete outcome data reported.	
	Age, mean years: 14		Total length: 1 year					
	Gender: 100% female							
	Context: Outpatient child and adolescent psychiatric clinic.							
	Mean illness duration: 2 years (range 1–4 years)							
Depestele et al. (2017), Belgium, Non‐randomised controlled clinical trial	112 adolescents (MFT = 62, MPT = 50)	One or more parents per patient. Participated. Further data not reported.	Multi‐family group with patient (MFT).	Multi‐parent group without patient (MPT). Comparator matched the intervention in all other ways.	At baseline and then post‐intervention at 8–9 weeks.	Eating disorder symptomatology (EDI), family functioning (FAD, all participants), familial burden (ECI, family).	MFT: 62/72 (86%) accepted invitation. 55/62 (89%) families randomised completed intervention. Post‐intervention data completeness ranged from 60% to 71%.	ED symptomatology (drive for thinness and body dissatisfaction) significantly improved across both interventions and independently of the presence of binge/purge behaviours. No significant effects found between interventions. Negative caregiving experiences significantly decreased across both interventions independent of presence/absence of binge/purge behaviour and independent of type of reporter. Family outcome measures showed significant improvements across family functioning according to the patients and fathers, but not to the mothers, across both interventions and ED subtypes.
	Diagnoses: AN restrictive subtype (41%), AN binge/purge subtype (23%), BN (21%), EDNOS (14%)		Total contact hours: 15	Total contact hours: 15			MPT: 51/79 (65%) accepted invitation. 45/51 (88%) families randomised completed intervention. Post‐intervention data completeness ranged from 50% to 77%.	
	Age, mean years: 17		Total length: 10 weeks	Total length: 10 weeks				
	Gender: 100% female							
	Context: Admitted to inpatient facility.							
Dimitropoulos et al. (2015), Canada, Non‐randomised controlled clinical trial	45 adults (MFT = 28, SFT = 17)	72 family members (36% mothers, 32% fathers, 21% partners, 11% siblings)	Multi‐family therapy (MFT).	Single family therapy (SFT). The purpose of SFT was to identify issues that were important to the patient to discuss with their family.	At baseline and then post‐intervention at 8 weeks, follow‐up 3 months later.	BMI, eating disorder symptomatology (EDE‐Q), family functioning (FQ, family), depression (BDI, family), familial burden (SPS, EDSIS, DCCFS, family).	MFT: 24/28 (86%) families randomised completed intervention.	No statistically significant differences between MFT and SFT: both forms of therapy resulted in significantly improved family outcomes on all measures and a significant increase in BMI. Individuals in an intensive treatment who participated in both family‐based interventions experienced improvements in eating behaviours and an increase in BMI, regardless of type of family therapy received.
	Diagnoses: AN restrictive subtype (44%),	Gender: 47% female	Total contact hours: 12	Total contact hours: 10–12			SFT: 13/17 (76%) families randomised completed intervention.	
	AN binge/purge subtype (56%)		Total length: 8 weeks	Total length: 8 weeks			Analyses conducted with 31 patients (82%) and 45 family members (74%). 46% of family members completed 3‐month follow‐up assessment.	
	Age, mean years: 26							
	Gender: 100% female							
	Context: Inpatient and day‐treatment programme.							
	Mean illness duration: 8 years (range 1–25 years)							
Eisler, Simic, Hodsoll, et al. (2016), UK, Multi‐site RCT	167 adolescents (MFT = 85, FT‐AN = 82)	70% came from ‘intact’ families. Further data not reported.	Multi‐family therapy for anorexia nervosa (MFT‐AN).	Family therapy for anorexia nervosa (FT‐AN). A manualised approach applied as published.	At baseline, 3 months mid‐intervention, post‐intervention at 12 months, and follow up at 18 months.	% Mean BMI, eating disorder symptomatology (Morgan/Russell global outcome scale, EDE‐Q), depression (BDI), self‐esteem (RSES), familial burden (ECI, family).	84/359 (23%) families approached declined participation in the study.	There was a statistically significant difference rating on the Morgan/Russell global outcome scale at 12 months, in favour of MFT‐AN: The odds of a good or intermediate outcome in the MFT‐AN group was 2.55 times higher than that of FT‐AN group. Adolescents in both treatment groups gained considerable weight over the course of treatment and follow‐up, though at 12 months there were no statistically significant differences between the two trial arms for % mean BMI, eating disorder psychopathology, depression or self‐esteem. However, at 18 months there was a significant difference in % mean BMI in favour of the MFT‐AN group.
	Diagnoses: AN (76%), EDNOS restrictive type (24%)		Total contact hours: Approx. 70 (10 days)	Total contact hours: The number and frequency of sessions is determined by clinicians, starting with weekly meetings, which are then gradually spread out to 3–4 weekly			MFT: 76/86 (88%) families randomised completed first 3 months of intervention.	
	Age, mean years: 16		Total length: 12 months	Total length: 12 months			FT‐AN: 73/83 (88%) families randomised completed first 3 months of intervention.	
	Gender: 91% female						53% of values for secondary outcome measures at follow‐up missing.	
	Ethnicity: <10% from non‐White background							
	Context: Outpatient specialist ED services in London.							
Gabel et al. (2014), Canada, Case‐control	50 adolescents (MFT + TAU = 25, matched TAU controls = 25)	Information on family members not reported.	Multiple family therapy (MFT).	TAU; medical monitoring, nutrition therapy, pharmacological treatment as needed, mental health therapy (combination of psychoeducation, and individual supportive family therapy), and inpatient admission if required.	Data acquired retrospectively. Data for weight and %IBW were collected from three time points: At assessment, prior to initiating MFT (parallel time for controls), and after completion of MFT (parallel time for controls). Both cases and controls completed psychometric self‐report measures at assessment.	%IBW, eating disorder symptomatology (EDI, EDE‐Q), depression (CDI).	N/A as retrospective audit of completers.	MFT group had a statistically significant higher percent healthy weight than TAU group. Measures of disordered eating symptoms and depression improved significantly after MFT, including significant improvements on the restraint, weight concerns and shape concerns subscales of the EDE‐Q, total EDE‐Q scores, and total depression scores.
	Diagnoses: AN (100%)		Total contact hours: Not reported	Total contact hours: Not reported			No incomplete outcome data reported.	
	Age, mean years: 14		Total length: Not specified though typically longer than 3–6 months.	Total length: Not reported				
	Gender: 100% female							
	Context: Multiple levels of care including inpatient, outpatient, and day hospital programmes.							
Gelin et al. (2015), Belgium, Case series	82 adolescents	70% came from ‘intact’ families.	Multiple family therapy (MFT).	No comparison group.	At baseline and then post‐intervention at 11 months.	%EBW, eating disorder symptomatology (EDI), psychological well‐being (OQ‐45).	7 (8.5%) families dropped out during treatment. 91.5% completed intervention.	At end of treatment, approximately half of patients achieved a %EBW above 85% and half remained within the clinical range. Patients showed significant changes on all EDI dimensions except for bulimia, though very few patients endorsed bulimic symptoms at baseline. 71% of patients achieved a score below clinical significance on the OQ‐45 and significant improvements in quality of life were found on all three OQ‐45 dimensions.
	Diagnoses: AN restrictive subtype (84%), AN binge/purge subtype (11%), BN (5%).		Total contact hours: Approx. 147 h (21 days)				Missing data ranged from 6% to 26% across outcome measures.	
	Age, mean years: 16		Total length: 11 months					
	Gender: 98% female							
	Context: Adolescent ED outpatient treatment centre.							
	Illness duration: 74% < 1 year							
Hollesen et al. (2013), Denmark, Case series	20 adolescents	Information on family members not reported.	Multiple family therapy (MFT).	No comparison group.	At baseline and then post‐intervention at 12 months.	BMI, eating disorder symptomatology (EDI, EDE‐Q), emotional and interpersonal difficulties (IIP, SASB‐Intrex).	1/32 (3%) families offered MFT dropped out of treatment. 97% completed intervention.	After MFT treatment, 13 patients (65%) were free of ED diagnosis. Significant improvement was found in BMI, restriction, eating concern, weight concern and amount of exercise after the MFT groups. Results from the EDI showed significant improvement with high effect sizes regarding drive for thinness and interoceptive awareness. Post‐treatment, patients reported less interpersonal problems on most IIP dimensions, but differences were not of statistical significance. No results regarding patients' relations to their parents reached statistical significance.
	Diagnoses: AN restrictive subtype (25%), AN binge/purge subtype (15%), EDNOS (60%)		Total contact hours: Approx. 84 (12 days)				11/32 (34%) families offered MFT did not complete outcome measures and were therefore excluded from analyses.	
	Age, mean years: 15		Total length: 12 months					
	Gender: 100% female							
	Context: Outpatient specialist ED service in Western Denmark.							
Marzola et al. (2015), USA, Retrospective cohort study	74 adolescents (M‐IFT = 54, S‐IFT = 20)	Information on family members not reported.	Intensive family therapy – Multiple families (M‐IFT).	Intensive family therapy – Single family (S‐IFT).	At baseline and then a mean of 30 months after the intervention.	%IBW, eating disorder symptomatology (EDE‐Q completed by patients and parents). Semi‐structured interview adapted from EDE‐Q.	All families (100%) completed full treatment.	At follow‐up, mean %IBW of the overall sample significantly increased, with a very large effect size. A significant increase in %IBW emerged in both diagnosis subgroups but no differences between S‐IFT and M‐IFT groups emerged.
	Diagnoses: AN (60%), EDNOS, restricting type (40%)		Total contact hours: 40	Total contact hours: 40			74/92 (80.5%) completed outcome measures for follow‐up study at 30 months.	
	Age, mean years: 15		Total length: 5 days	Total length: 5 days				
	Mean illness duration: 2 years							
	Gender: 92% female							
	Ethnicity: 92% Caucasian							
	Context: Treatment completed between Nov 2006 and Jun 2013 at services linked with University of California, San Diego.							
Mehl et al. (2013), Czech Republic, Case series	15 adolescents and young adults	Information on family members not reported.	Multi‐family therapy (MFT).	No comparison group.	At baseline and then post‐intervention at 12 months.	BMI, self‐esteem (RSES), psychological well‐being (SOS‐10).	2 families (13%) dropped out (1 patient and 1 entire family). 87% completed intervention.	Patients experienced significant improvements in quality of life after MFT, though a significant reduction in self‐esteem. Patients' BMIs significantly improved during MFT, which the authors argue may explain the decrease in self‐esteem.
	Diagnoses: Not specified		Total contact hours: Approx. 64 (8 days)				All 15 patients (100%) completing intervention completed outcome measures.	
	Age, mean years: 18		Total length: 12 months					
	Gender: Not specified							
	Context: Outpatient and inpatient specialist clinic in Prague. All patients lived at home with their families.							
Salaminiou et al. (2017), UK, Case series	30 adolescents	29 mothers, 22 fathers. 73% came from ‘intact’ families.	Intensive multi‐family therapy (MFT).	No comparison group.	At baseline, 3 months into the intervention, and 6 months into the intervention.	% Mean BMI, eating disorder symptomatology (Morgan/Russell global outcome scale, EDI‐II), self‐esteem (RSES), depression (BDI‐II patients and family members).	2 families (7%) discontinued intervention. 93% completed intervention.	Patients' weight, eating disorders' psychopathology, mood and self‐esteem significantly improved over the 6 months of the study. At 3 months, half the patients were classified as having reached an intermediate or good outcome on the Morgan/Russell global outcome scale. At 6 months, just over one‐third of patients were classified as having a poor outcome but the remaining could be classified as partially remitted. Parents' self‐rating of mood improved over the course of the 6 months: The improvement was significant for the mothers but not for the fathers, though scores were in the normal range before treatment.
	Diagnoses: AN (90%), EDNOS, restricting type (3%)		Total contact hours: Approx. 77 (9–11 days)				26/30 (87%) patients and families completed outcome measures.	
	Age, mean years: 15		Total length: 9 months					
	Gender: 90% female							
	Mean illness duration: 12 months							
	Context: Outpatient specialist ED services in London.							
Skarbø and Balmbra (2020), Norway, Case series	68 young adults	198 family members have participated: 65 mothers, 56 fathers, 59 siblings, 12 partners, 6 other relatives.	Multifamily therapy (MFT).	No comparison group.	At baseline and then post‐intervention at 12 months.	BMI	5 (7%) families dropped out before completing intervention. 93% completed intervention.	BMI significantly increased over the course of treatment for those who were underweight at the start.
	Diagnoses: AN (76.5%), BN (23.5%)		Total contact hours: Approx. 91 (13 days)				No outcome measures completed. 55/68 (81%) had post‐intervention BMI data.	
	Age, mean years: 21		Total length: 12 months					
	Gender: 100% female							
	Context: Outpatient setting							
Stewart et al. (2021), UK, Case series	50 adolescents	Information on family members not reported.	Multi‐family therapy for bulimia nervosa (MFT‐BN).	No comparison group.	At baseline and then post‐intervention at 4 months.	Eating disorder symptomatology (EDE‐Q), anxiety (RCADS child, RCADS family, HADS family), depression ((RCADS child, RCADS family, HADS family), emotional and interpersonal difficulties (DERS), familial burden (ECI, family)	N/A as retrospective audit of completers.	Adolescents reported significant reductions in eating disorder symptoms including shape concern, weight concern, and frequency of binge/purge episodes. Adolescents also reported significant improvements in anxiety and depression and significant reductions in emotion regulation difficulties. Parents did not report significant changes in their child's depression or anxiety though did report significant reductions in their own depression symptoms and significant reductions in negative caregiving experiences.
	Diagnoses: BN (100%)		Total contact hours: 28				28/50 (56%) of patients completed pre‐ and post‐EDE‐Q assessments.	
	Age, mean years: 16		Total length: 4 months					
	Gender: 98% female							
	Context: Outpatient specialist ED service in London, treatment completed between Sep 2009 and Dec 2018.							
Tantillo et al. (2019), USA, Case series	10 young adults	Information on family members not reported.	Reconnecting for recovery (R4R) multifamily therapy group for anorexia nervosa (MFTG).	No comparison group.	At baseline, post‐intervention at 26 weeks, and 6‐month follow‐up.	BMI, eating disorder symptomatology (EDE‐Q), emotional and interpersonal difficulties (DERS).	No premature drop‐outs reported, all families (100%) attended ≥13 sessions.	There were clinically and statistically significant improvements in ED symptomatology and emotion regulation difficulties from baseline to end of treatment. This trajectory of improvement in these measures further continued at the 6‐month follow‐up. BMI increased though changes were modest.
	Diagnoses: OSFED (including atypical AN) (60%), AN (40%)		Total contact hours: 24				No incomplete outcome data reported.	
	Age, mean years: 23		Total length: 26 weeks					
	Gender: 100% female							
	Mean illness duration: 8 years							
	Context: Recruited from the community in New York state. 70% lived at home with family.							
Whitney et al. (2012), UK, Single‐site RCT	48 adults (FDW = 25, IFW = 23)	119 family members/carers: FDW = 65, IFW = 54. Across groups 39 mothers, 36 fathers, 44 significant others.	Family day workshops (FDW).	Individual family work (IFW).	Patient assessments completed on admission to unit, at discharge, and 3‐year follow‐up. Family members assessments completed at baseline, 6‐month follow up, and 3‐year follow‐up.	BMI, eating disorder symptomatology (SEED), emotional and interpersonal problems (IIP), psychological well‐being (GHQ‐12, family), family functioning (LEE, family), familial burden (ECI, family).	22/25 (88%) of families in FDW group received intervention. 23/25 (92%) patients completed primary outcomes; 17/25 (68%) completed secondary outcomes. 44/67 (66%) of carers completed long‐term assessments.20/23 (87%) of families in IFW group received intervention. 21/23 (91%) patients completed primary outcomes; 16/23 (70%) completed secondary outcomes. 38/58 (66%) of carers completed long‐term assessments.	Patients had significant and persistent improvements in their BMI, and carers had a reduction in their stress level; however, no clinically significant differences between the two forms of intervention were found in the outcomes of either patient or carer.
	Diagnoses: AN (100%)		Total contact hours: Approx. 21	Total contact hours: 18				
	Age, mean years: Not reported		Total length: 3 days	Total length: Variable, 1–2‐h sessions provided weekly or fortnightly, plus three follow‐up sessions.				
	Gender: 98% female							
	Median illness duration: 5–10 years							
	Context: Specialist inpatient ED units in London.							
Wierenga et al. (2018), USA, Case series	55 adults	73 family members/carers: Parents: 36 mothers, 17 fathers, 20 other family members or significant others. 39 (53%) patients were accompanied by 1 support, 14 (19%) patients by 2 supports, and 2 (3%) patients by three supports.	Neurobiologically‐informed 5‐day multi‐family treatment for AN.	No comparison group.	At baseline, post‐intervention at 5 days, and 3‐month follow‐up.	BMI, %IBW, eating disorder symptomatology (EDE‐Q, patients and family members), family functioning (FAD), anxiety (STAI).	1 family (2%) did not complete treatment as higher level of intervention was required. 98% completed intervention.	BMI significantly increased after the 5‐day intervention, but it is noted the change may not be clinically meaningful. Patients reported significant reductions in eating disorder symptomatology and state anxiety as well as improvements in family functioning. Data from family members and supports who completed pre‐ and post‐treatment measures (reflecting 50 patients) showed significant improvements in observed ED symptomatology and family functioning. Post‐treatment, 10% of patients were classified as fully remitted, 21% as partially remitted, and 69% reported a poor outcome. At follow‐up, 62% of patients achieved either full remission or partial remission, while 38% reported a poor outcome.
	Diagnoses: AN restrictive subtype (52%), AN binge/purge subtype (24%) EDNOS restrictive subtype (4%), AN partial remission (20%)		Total contact hours: 40				50/54 (93%) of patients completed post‐treatment assessments. 27/54 (50%) patients completed follow‐up assessments.	
	Age, mean years: 25		Total length: 5 days				31% of supports completed follow‐up assessments.	
	Gender: 100% female							
	Mean illness duration: 9 years							
	Context: Recruited via clinic websites of ED clinics in CA and OH.							

*Note*: %EBW, percentage expected body weight; %IBW, percentage ideal body weight; AN, anorexia nervosa; BDI, Beck Depression Inventory; BMI, body mass index; BN, bulimia nervosa; CDI, children's depression inventory; CGAS, children's global assessment scale; DCCFS, devaluation of consumers and consumer families questionnaire; DERS, difficulties in emotion regulation scale; ECI, experience of caregiving inventory; ED, eating disorder; EDE‐Q, eating disorder examination questionnaire; EDI, Eating Disorder Inventory; EDNOS, eating disorder not otherwise specified; EDSIS, eating disorder impact scale; FAD, family assessment device; FQ, family questionnaire; GHQ‐12, general health questionnaire 12; HADS, hospital anxiety and depression scale; IIP, inventory of interpersonal problems; LEE, perceived level of expressed emotion; OQ‐45, outcome questionnaire 45; OSFED, other specified feeding or eating disorder; RCADS, revised children's anxiety and depression scale; RSES, Rosenberg's self‐esteem scale; SASB‐Intrex, structural analysis of social behaviour; SEED, short evaluation of eating disorders; SOS‐10, Schwartz outcome scale 10; SPS, social provisions scale; STAI, Spielberger state‐trait anxiety inventory (STAI); TAU, treatment as usual.

### 2.4 Quality assessment of studies

The methodological quality of all included studies was assessed using the Effective Public Healthcare Panacea Project (EPHPP) quality assessment tool (National Collaborating Centre for Methods and Tools [Internet], 2008). The EPHPP quality assessment tool evaluates eight components: selection bias, study design, confounders, blinding, data collection methods, withdrawals or drop‐outs, intervention integrity and analyses. A rating of ‘strong’, ‘moderate’ or ‘weak’ was assigned to each category and a global rating calculated based on the frequency of weak ratings across these components. In accordance with Cochrane guidance for randomised trials, version two of the Cochrane risk‐of‐bias tool for randomised trials (RoB 2) was used. The RoB 2 gives a judgement of ‘low risk’, ‘high risk’ or ‘some concerns’ of specific bias domains based on the responses to signalling questions. A study's overall risk of bias is the least favourable assessment across all domains.

The risk of bias and methodological quality was determined by one reviewer (JZ) and a sub‐selection (33%) of the papers were independently rated by a second reviewer (NOD). Decisions were compared and discussed to achieve a consensus on any discrepancies.

### 2.5 Data analysis and synthesis

All outcomes were measured as continuous data. End point scores were expressed as mean differences (MDs) or standardised mean differences (SMDs) with 95% confidence intervals (CIs). Heterogeneity of the studies was assessed by visual inspection of forest plots and calculation of the *I* ^2^ statistic using RevMan 5.4.1 (Review Manager, 2020). An *I* ^2^ up to 25% indicates low heterogeneity; up to 50% indicates moderate heterogeneity; and 75% or greater, high heterogeneity. Investigation of heterogeneity was not performed as a minimum of 10 studies are required for subgroup analyses (Higgins & Green,  2011). The meta‐analysis used random effect models if *I* ^2^ > 50%, and fixed effects if *I* ^2^ < 50%. When higher scores on a measure represented better outcomes, forest plots were constructed with intervention data on the right. When lower scores represented better outcomes, intervention data were presented on the left. As recommended, when different scales measuring the same construct differed in the direction that represents a better outcome, the mean was subtracted from the maximum possible value for the scale for differing studies to ensure all scales pointed in the same direction (Higgins et al., 2021). Standard deviations remained the same. Studies were categorised as having a short‐term follow‐up (up to and including 3 months after baseline), medium‐term follow‐up (3–12 months after baseline), or long‐term follow‐up (beyond 12 months after baseline). In pre/post analyses, the longest follow‐up timepoint available was always used. A narrative synthesis was conducted where meta‐analysis was not appropriate. However, as a detailed narrative synthesis of studies has been recently provided in the scoping review by Baudinet et al. (2021), this information has not been repeated in this article.

## 3 RESULTS

### 3.1 Study selection

**FIGURE 1:**
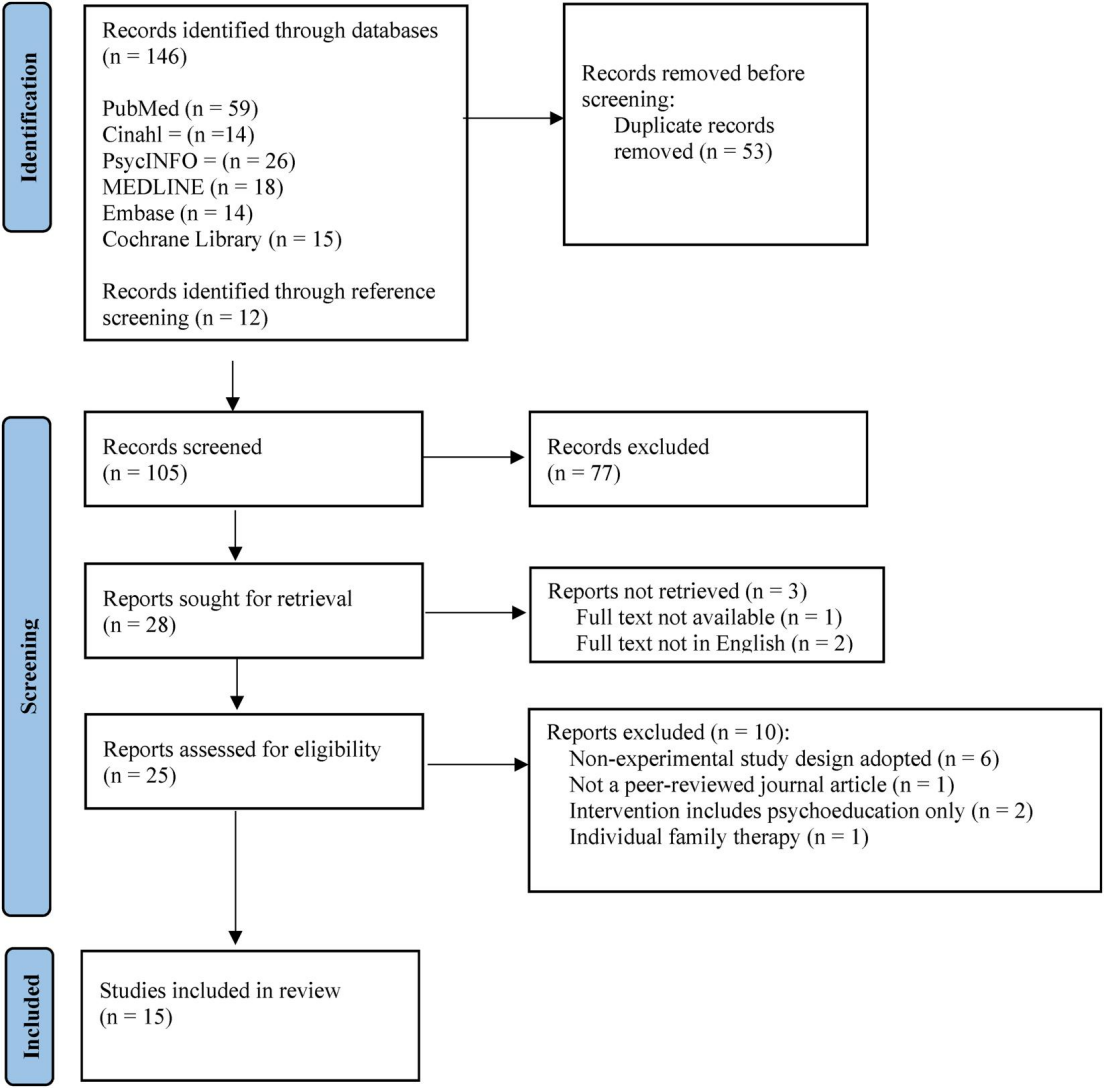
PRISMA diagram displaying process for study selection

The search strategy identified 105 studies for possible inclusion; 77 were excluded at the title or abstract level and the full texts of 25 studies were accessed to determine eligibility (Figure 1). The full English texts of three papers could not be retrieved. 15 studies met all inclusion criteria. These were reviewed for study design, patient and family characteristics, interventions, and outcomes and the relevant data entered into Review Manager 5.4.1 (Review Manager, 2020).

### 3.2 Study characteristics

The 15 studies included two RCTs, two controlled trials, nine case series, one retrospective cohort study, and one case control study. Studies were published between 2012 and 2021. Studies were conducted in either North America (five studies) or Europe (10 studies), with four of these from the UK. Five studies trialled MFT for adults with EDs (one in the inpatient setting, three in the outpatient setting, one across both settings), eight studies researched adolescents (one in the inpatient setting, six in the outpatient setting and one across both) and one study researched both adolescents and young adults in the outpatient setting (Table 1). The interventions ranged in length from three days to 12 months; contact hours with healthcare professionals ranged from approximately 12 to 147, when this was reported. Follow‐up periods varied between immediate post‐intervention assessment and 3 years (Table 2).

**TABLE 2:** Summary of intervention characteristics

Reference and study design	Intervention name	Model(s) of therapy	Intervention structure and length	Adjunct versus sole therapy
Dennhag et al. (2021)	Multi‐family therapy (MFT)	Wallin (2007)	Four‐day intensive programme followed by six and a half days of treatment over 1 year.	Adjunct to TAU (single family therapy, individual therapy, or inpatient treatment).
Case series				
Depestele et al. (2017)	Multi‐family group with patient (MFT)	Maudsley model (Eisler, 2005). Intervention described in Depestele et al. (2015)	One three‐hour introduction session, five sessions every 2 weeks and one follow‐up session after 6 months. Siblings excluded.	Adjunct to inpatient treatment.
Non‐randomised controlled clinical trial				
Dimitropoulos et al. (2015)	Multi‐family therapy (MFT)	Cognitive‐interpersonal maintenance model of AN (Schmidt & Treasure, 2006; Treasure & Schmidt, 2013)	Weekly 90‐min sessions for 8 weeks.	Adjunct to inpatient and/or day patient treatment.
Non‐randomised controlled clinical trial				
Eisler et al. (2016b)	Multi‐family therapy for anorexia nervosa (MFT‐AN)	Maudsley model (Dare & Eisler, 2000; Simic & Eisler, 2012) based on FT‐AN (Eisler et al., 2015)	Four‐day intensive programme followed by an additional six 1‐day meetings at 4‐ to 8‐week intervals over 9 months.	Adjunct to treatment already being received in National health service.
RCT				
Gabel et al. (2014)	Multiple family therapy (MFT)	Maudsley model (Dare & Eisler, 2000)	Not specified.	Adjunct to TAU for cases (medical monitoring, nutrition therapy including meal plans, pharmacological treatment as required, mental health therapy (a combination of psychoeducation, and individual supportive family therapy), and inpatient admission if required.
Case‐control				
Gelin et al. (2015)	Multiple family therapy (MFT)	Developed using Maudsley model (Dare & Eisler, 2000) and Dresden model (Scholz & Asen, 2001)	21 days delivered over 11 months, organised in blocks of days.	Not specified.
Case series				
Hollesen et al. (2013)	Multiple family therapy (MFT)	Developed using Maudsley model (Dare & Eisler, 2000) and Dresden model (Scholz & Asen, 2001)	12 days spread over approximately 1 year.	Adjunct to TAU (outpatient family therapy and individual therapy).
Case series				
Marzola et al. (2015)	Intensive family therapy ‐ multiple families (M‐IFT)	Incorporates FT‐AN, structural, strategic, systemic, narrative and psychodrama‐based family approaches	5 consecutive days.	Sole therapy.
Retrospective cohort study				
Mehl et al. (2013)	Multi‐family therapy (MFT)	Maudsley model (Dare & Eisler, 2000)	Three‐day initial block followed by monthly follow‐up days for 5 months.	Adjunct to inpatient treatment.
Case series				
Salaminiou et al. (2017)	Intensive multi‐family therapy (MFT)	Maudsley model (Dare & Eisler, 2000)	Four‐day multi‐family programme followed by an additional 5 days over 9 months.	Adjunct: Depending on families' needs, they may have been seen individually between meetings.
Case series				
Skarbø and Balmbra (2020)	Multifamily therapy (MFT)	Maudsley (Treasure et al., 2012) and Toronto models (Dimitropoulos et al., 2015) for adults	6 group meetings (totalling 13 days) spanning 12 months.	Adjunct to follow‐ups from their own local therapist.
Case series				
Stewart et al. (2021)	Multi‐family therapy for bulimia nervosa (MFT‐BN)	Systemic therapy in a multi‐family context (Simin & Eisler, 2015), with cognitive behavioural therapy and dialectical behavioural therapy	2 h weekly over a period of 4 months, providing up to 28 h of treatment.	Adjunct to existing BN approaches.
Case series				
Tantillo et al. (2019)	Reconnecting for recovery (R4R) multifamily therapy group for anorexia nervosa (MFTG)	Relational and motivational manualised intervention (Tantillo et al., 2015)	16 90‐min sessions over 26 weeks.	Adjunct to out‐patient treatment (if this was already being received).
Case series				
Whitney et al. (2012)	Family day workshops (FDW)	Family psychoeducation and skills training workshops (Treasure et al., 2012)	3 consecutive days.	Adjunct to inpatient treatment.
RCT				
Wierenga et al. (2018)	Neurobiologically‐Informed 5‐day multi‐family treatment for AN	Intensive temperament‐ and neurobiologically‐based therapy (Kaye et al., 2015)	5 consecutive days.	Adjunct to other treatments received in and beyond the service (though not specified).
Case series				

*Note*: TAU, treatment as usual.

One study (Depestele et al., 2017) was categorised as having short‐term outcomes, 10 were categorised as having medium‐term outcomes (Dennhag et al., 2021; Dimitropoulos et al., 2015; Gelin et al., 2015; Hollesen et al., 2013; Mehl et al., 2013; Salaminiou et al., 2017; Skarbø & Balmbra, 2020; Stewart et al., 2021; Tantillo et al., 2019; Wierenga et al., 2018), and three were categorised as having long‐term outcomes (Eisler et al., 2016b; Marzola et al., 2015; Whitney et al., 2012). Six studies utilised a comparison group (Depestele et al., 2017; Dimitropoulos et al., 2015; Eisler et al., 2016b; Gabel et al., 2014; Marzola et al., 2015; Whitney et al., 2012). No studies had more than two arms. Attempts to obtain the additional data needed to include all studies in the meta‐analysis were unsuccessful as not all authors were contactable or able to provide the required information. All studies measured patient outcomes using either physical (e.g., weight) or psychosocial (e.g., questionnaires measuring mood and ED symptoms) assessments and seven measured psychosocial outcomes in family members (Depestele et al., 2017; Dimitropoulos et al., 2015; Eisler et al., 2016b; Marzola et al., 2015; Salaminiou et al., 2017; Stewart et al., 2021; Whitney et al., 2012).

### 3.3 Patient characteristics

A total of 850 patients were included; sample sizes ranged from 10 (Tantillo et al., 2019) to 167 (Eisler et al., 2016b), with the majority of studies (13; 87%) having a sample size of less than 85 participants. Gender was reported in 14 of 15 studies and the percentage of each sample population noted to be female ranged from 90% to 100%. The mean age of patients included in each study ranged from 14 (Gabel et al., 2014) to 26 years (Dimitropoulos et al., 2015). With regard to ED diagnosis, five studies included patients with restrictive EDs only, primarily AN‐restricting with a minority with an EDNOS‐restricting diagnosis (Eisler et al., 2016b; Gabel et al., 2014; Marzola et al., 2015; Salaminiou et al., 2017; Whitney et al., 2012). Eight studies recruited patients with a range of ED diagnoses which included combinations of AN‐restricting, AN binge/purge, EDNOS, OSFED, atypical AN, and BN (Dennhag et al., 2021; Depestele et al., 2017; Dimitropoulos et al., 2015; Gelin et al., 2015; Hollesen et al., 2013; Skarbø & Balmbra, 2020; Tantillo et al., 2019; Wierenga et al., 2018). One study evaluated MFT only amongst patients with a diagnosis of BN (Stewart et al., 2021).

Information on participating family members was reported in nine studies. Two of these studies reported on family structures where 73% (Salaminiou et al., 2017) and 70% (Eisler et al., 2016b) came from ‘intact’ families. Six studies described the demographics of family members (Table 1). Mothers made up the largest proportion of participating family members ranging from 32% (Whitney et al., 2012) to 57% (Salaminiou et al., 2017) of the described sample.

### 3.4 Risk of bias

Quality appraisals are presented in Tables S1 and S2 (Supplementary Material). All studies were appraised using the EPHPP. The two RCTs (Eisler et al., 2016b; Whitney et al., 2012) received ‘strong’ global ratings eight were rated as ‘moderate’ and five were rated as ‘weak’. Given the barriers to blinding in psychological intervention research, most studies received lower quality ratings due to the lack of assessor and participant blinding. Studies given a global rating of ‘weak’ were most often deemed so when blinding issues were combined with validity and reliability of data collection methods not being reported, or higher withdrawal rates. Using the RoB 2 tool, both RCTs were identified has having ‘some concerns’ due to potential bias in the randomisation process and/or selection of the reported result.

### 3.5 Meta‐analytic findings

#### 3.5.1 Objective 1: Patient's weight & ED symptomatology

**FIGURE 2:**
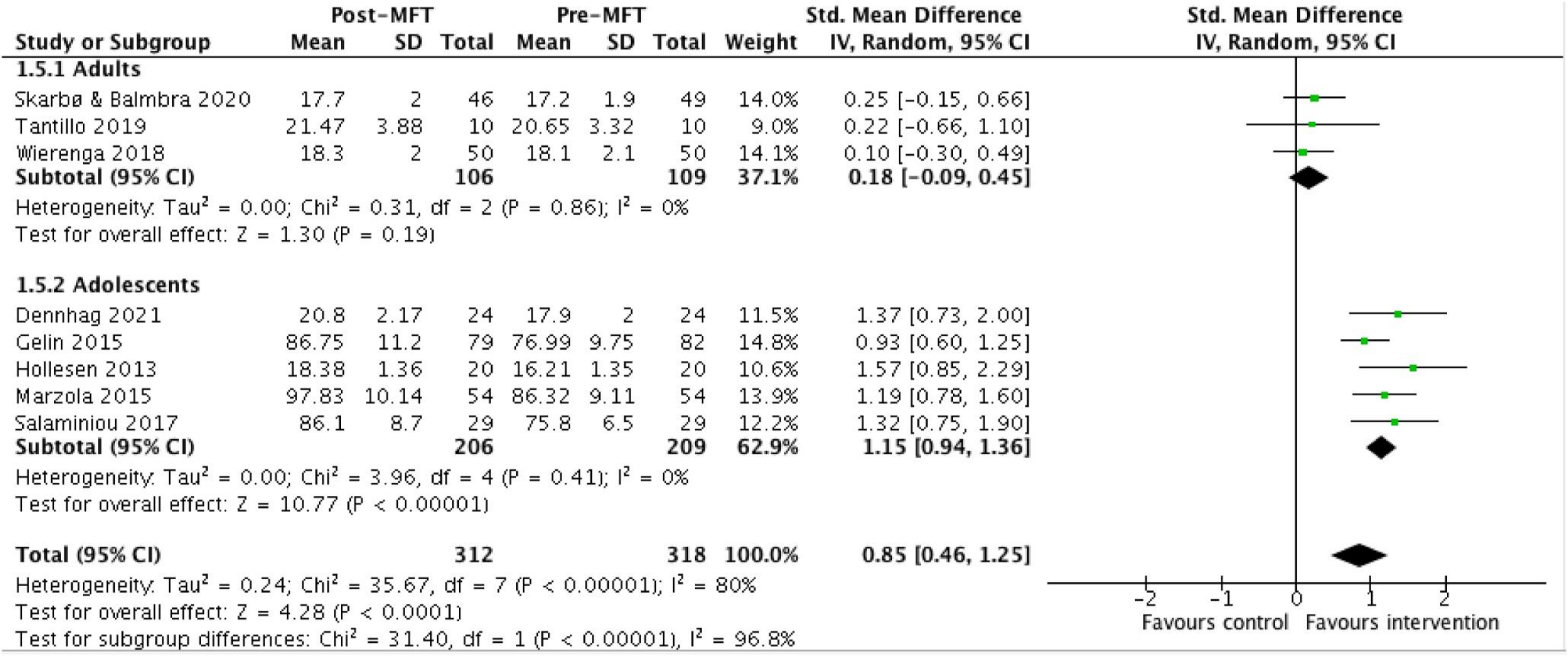
Table and forest plot of pooled data comparing the change in weight before and after receiving MFT

Four comparison studies reported data that was suitable to be pooled for weight (*n* = 283) (Eisler et al., 2016b; Gabel et al., 2014; Marzola et al., 2015; Whitney et al., 2012). The evidence showed no significant improvement on weight compared to control interventions (SMD = 0.12, 95% CI = −0.42, 0.67). Eight additional studies reported pre‐post data that was suitable to be pooled for this outcome (*n* = 676; Dennhag et al., 2021; Gelin et al., 2015; Hollesen et al., 2013; Marzola et al., 2015; Salaminiou et al., 2017; Skarbø and Balmbra, 2020; Tantillo et al., 2019; Wierenga et al., 2018). Overall, there was a significant increase in patients' weight across studies, with a large effect size (SMD = 0.85, 95% CI = 1.25, 0.46; Figure 2). When split into subgroups of studies assessing MFT amongst adults as compared to amongst adolescents, there is evidence of significant improvements in weight of a large effect size amongst adolescents (SMD = 1.15, 95% CI = 0.94, 1.36), but not amongst adults (SMD = 0.18, 95% CI = −0.09, 0.45).

**FIGURE 3:**
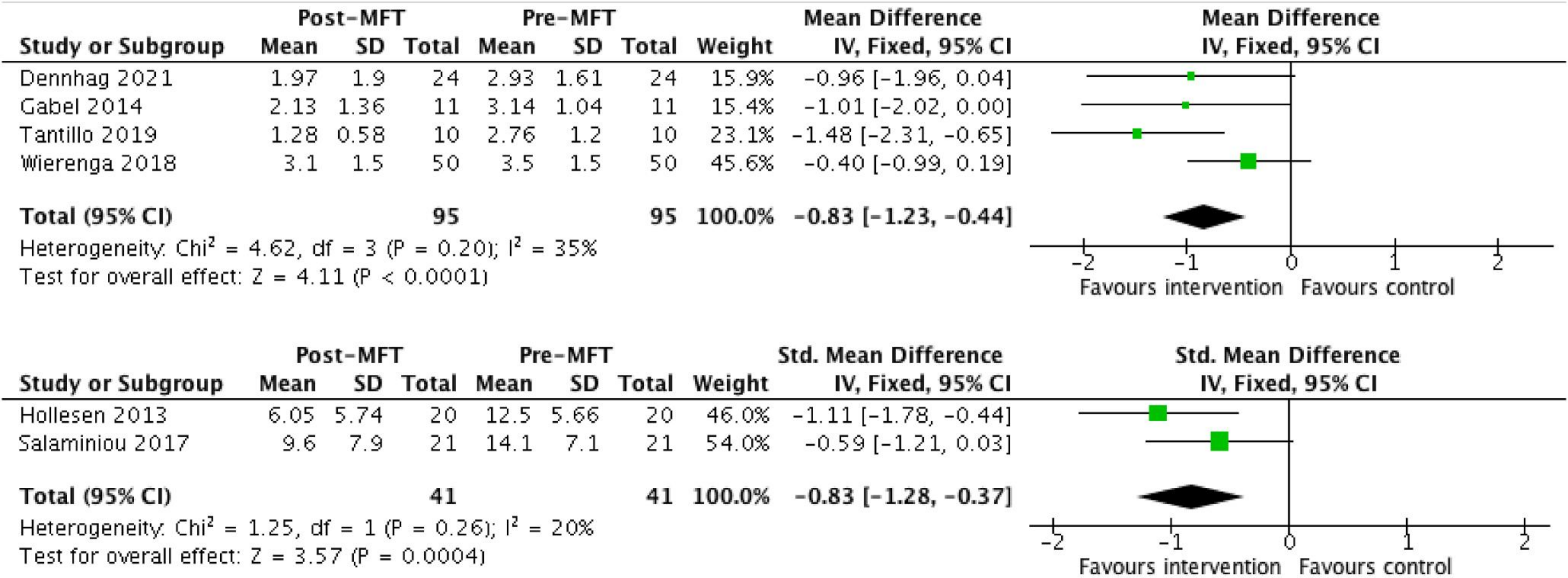
Tables and forest plots of pooled data comparing the change in self‐reported ED symptoms (top) and AN‐specific symptoms before and after receiving MFT (bottom)

Two comparison studies reported data on patient's ED symptomatology that could be pooled (*n* = 125; Eisler et al., 2016b; Marzola et al., 2015). Both studies included data from the EDE‐Q and reported global scores and subscale scores. There were no significant differences in changes in self‐reported ED symptoms as measured by the EDE‐Q (global score and its four subscales, restraint, eating concern, shape concern, weight concern) between MFT and comparison groups (FT‐AN; Eisler et al., 2016b) and an intensive 5‐day single family therapy (Marzola et al., 2015). Four additional studies reported EDE‐Q global scores before and after the MFT intervention (*n* = 190; Wierenga et al., 2018; Tantillo et al., 2019; Dennhag et al., 2021; Gabel et al., 2014). A significant improvement of medium effect size was found in self‐reported ED symptoms after an MFT intervention (MD = −0.83, 95% CI = −1.23, −0.44; Figure 3). The study with the largest effect size (Tantillo et al., 2019) evaluated a 26‐week long intervention for adults.

With regard to AN symptoms specifically, results from the SEED AN subscale and drive for thinness subscale from the EDI were pooled as these two subscales have shown to be significantly positively correlated in ED patients (Bauer et al., 2005). Two comparison studies collected data suitable to be pooled in for anorexia symptoms (*n* = 91; Depestele et al., 2017; Whitney et al., 2012). No significant change was found in symptoms of anorexia between the MFT and control group across these two studies (SMD = 0.22, 95% CI = −0.20, 0.63). An additional two studies collected pre‐post data for anorexia symptoms (*n* = 82; Hollesen et al., 2013; Salaminiou et al., 2017). A significant improvement in self‐reported anorexia symptoms was found after the MFT intervention, with a large effect size (SMD = 0.83; 95% CI = 0.37, 1.28). Both studies evaluated outcomes in the medium‐term and studied adolescents with AN and EDNOS diagnoses.

#### 3.5.2 Objective 1: Patient's mood & psychological well‐being

Four studies reported suitable pre‐post data to be pooled for patient's depression (*n* = 214; Eisler et al., 2016b; Gabel et al., 2014; Salaminiou et al., 2017; Stewart et al., 2021). Overall, there was a significant decrease in patients' report of depression symptoms across studies, with a medium effect size (SMD = −0.59, 95% CI = −0.88, −0.31). Significant improvements were seen across diagnoses in adolescents with BN (Stewart et al., 2021) and restrictive EDs (Salaminiou et al., 2017) Three studies reported pre‐post data that could be reported for patient's general psychological well‐being (*n* = 222; Dennhag et al., 2021; Gelin et al., 2015; Mehl et al., 2013). Overall, there was a significant increase in patients' general psychological well‐being, with a large effect size (SMD = 1.09, 95% CI = 0.81, 1.38). Forest plots for these outcomes found in Supplementary Material (Figure S1).

#### 3.5.3 Objective 1: Family members' mood and well‐being

Two comparison studies, both of which recruited adult patients, reported data that was suitable to be pooled for this outcome (*n* = 114; Dimitropoulos et al., 2015; Whitney et al., 2012). No significant improvement was found in the MFT groups compared to control (SMD = 0.22, 95% CI = −0.15, 0.60). Two additional studies, both of which recruited adolescent patients, reported pre‐post data that could be pooled for this outcome (*n* = 114; Salaminiou et al., 2017; Stewart et al., 2021). Overall, there was a significant reduction in parents' self‐reported depression symptoms, of a medium effect size (SMD = −0.47, 95% CI = −0.84, −0.10) with a larger effect size reported in a sample of patients with AN (Salaminiou et al., 2017) compared to BN (Stewart et al., 2021). Forest plot found in Supplementary Material (Figure S2).

#### 3.5.4 Objective 2: Family functioning and caregiver burden

Two studies reported pre‐post data that was suitable to be pooled to assess family functioning, as reported by family members (*n* = 229; Depestele et al., 2017; Wierenga et al., 2018). Overall, no significant improvement was found on this outcome after as compared to before the intervention (SMD = −0.3, 95% CI = −0.78, 0.18). However, in isolation, a significant improvement in family functioning was found amongst adult patients (Wierenga et al., 2018).

With regard to negative appraisals of caregiving, four comparison studies reported data that was suitable to be pooled for this outcome (*n* = 274; Depestele et al., 2017; Dimitropoulos et al., 2015; Eisler et al., 2016b; Whitney et al., 2012). Depestele et al. (2017) gathered separate data for on the ECI (positive and negative subscales) for mothers and fathers. Both datasets could not be included in meta‐analysis, nor combined, therefore data for mothers only were included in the meta‐analysis as they formed a larger sample and other studies also included more mothers than fathers. The evidence showed no significant improvement on negative caregiving appraisals compared to control interventions (SMD = 0.06, 95% CI = −0.18, 0.30). Two additional pre‐post studies included data from adolescent patients that could be pooled from this outcome (*n* = 116; Dennhag et al., 2021; Stewart et al., 2021). These studies found a significant reduction in parental burden of a medium effect size (SMD = −0.58, 95% CI = −0.95, −0.20), though when looking at AN patients exclusively, the effect size was large (Dennhag et al., 2021). Forest plot found in Supplementary Material (Figure S3).

With regard to positive experiences of caregiving and support, four comparison studies reported data that was suitable to be pooled for this outcome, including adolescent and adult AN patients (*n* = 257; Depestele et al., 2017; Dimitropoulos et al., 2015; Eisler et al., 2016b; Whitney et al., 2012). The mothers only data were entered into RevMan as described above. The data also showed no significant change on positive caregiving experiences compared to control interventions (SMD = 0.05, 95% CI = −0.18, 0.29). No additional pre‐post studies had data that could be pooled for this outcome, however subgroup analyses of adults versus adolescent samples could be conducted. Neither data on solely adolescent samples (SMD = 0.00, 95% CI = −0.30, 0.30) nor adult samples (SMD = 0.15, 95% CI = −0.24, 0.53) demonstrated any significant improvements in positive caregiving experiences after MFT.

### 3.6 Review findings

Since the prospective publication of this systematic review and meta‐analysis’ protocol, a scoping review of quantitative findings of studies assessing the impact of MFT on patient's ED symptomatology, has been conducted by Baudinet et al. (2021) and as such, only a brief summary of these reports are provided here in line with our published protocol.

To summarise, five articles reported changes on patient's ED symptomatology which could not be included in the meta‐analysis (Dimitropoulos et al., 2015; Gelin et al., 2015; Marzola et al., 2015; Stewart et al., 2021; Wierenga et al., 2018). There were significant improvements in ED symptoms for both AN and BN patients (Dimitropoulos et al., 2015; Gelin et al., 2015; Stewart et al., 2021; Wierenga et al., 2018) including improvements in symptoms as reported by parents (Wierenga et al., 2018), however the studies utilising comparison group did not find any significant differences in ED symptom improvement between patients receiving MFT and those receiving the comparison intervention (Dimitropoulos et al., 2015; Marzola et al., 2015). With regard to comorbidities, significant improvements were also seen in self‐reported levels of anxiety (Stewart et al., 2021), self‐reported state anxiety (Wierenga et al., 2018), though this did not extend to trait anxiety (Wierenga et al., 2018) nor parents' own levels of anxiety (Stewart et al., 2021). Significant reductions were also found in levels of expressed emotion (Whitney et al., 2012) and criticism, over‐involvement, familial burden, and availability of social support (Dimitropoulos et al., 2015) however similar improvements were seen with MFT as compared to control therapies.

#### 3.6.1 Objective 1: Comparison of findings between adolescent and adult patients

Four of the papers which recruited adult patients (Dimitropoulos et al., 2015; Skarbø and Balmbra, 2020; Tantillo et al., 2019; Wierenga et al., 2018) targeted young adults with mean sample ages ranging from 18 to 26 years. Whitney et al. (2012) did not specify the age of their sample and Mehl et al. (2013) recruited both adolescents and young adults. Meta‐analyses were only conducted comparing adolescent and adult samples for changes in weight and changes to positive caregiving experiences pre‐to post‐MFT. With regard to patient's weight, there was evidence of significant improvements of a large effect size amongst adolescents, but no significant improvement was found amongst adults. With regard to positive experiences of caregiving, neither data on solely adolescent sample nor adult samples demonstrated any significant improvements after receiving MFT.

Additional meta‐analyses were not conducted due to too few studies preventing such subgroup analysis, thus the remaining data were narratively synthesised. Taken as a whole, papers looking at the effects of MFT on adolescents versus young adults report similar findings: trends towards improvements in ED symptoms, negative experiences of caregiving, and carer burden, though the significance of these trends varied. Of the studies which compared MFT to another intervention, therefore using a superiority approach, two studies had an adult sample and four had an adolescent sample. Whilst all reported significant improvements in BMI and/or other measurements of ED symptoms, only two of the studies reported significant differences between groups in favour of MFT (Eisler et al., 2016b; Gabel et al., 2014), both of whom recruited adolescents.

#### 3.6.2 Objective 3: Acceptability and retention rates

Treatment completion and retention rates were reported by all studies except those which retrospectively gathered cohort data (Gabel et al., 2014; Stewart et al., 2021). Two studies reported the percentage of participants accepting the invitation to participate in a research study. Depestele et al. (2017) found the acceptance rate was higher for those offered MFT (86%) compared to those offered the comparison parent‐only intervention (65%) and Eisler et al. (2016b) found 77% of all families approached accepted the invite to participate.

14 studies reported the proportion of participants completing MFT, with two studies reporting no premature dropouts (Marzola et al., 2015; Tantillo et al., 2019). The lowest completion rate reported was 86% (Dimitropoulos et al., 2015). When looking at the interventions that were deemed at least medium‐term (longer than 3 months), completion rates remained high and ranged from 100% (Tantillo et al., 2019) to 88% (Eisler et al., 2016b). Of the six studies which utilised a comparison group, five had completion data available. Marzola et al. (2015) reported 100% of randomised families completed their 5‐day intervention. For interventions deemed at least medium term (either a multi‐parent group or single‐family therapy, completion rates ranged from 88% (Depestele et al., 2017; Eisler et al., 2016b) to 76% (Dimitropoulos et al., 2015).

Completion of questionnaires at the end of treatment and follow‐up timepoints was reported in 12 studies and was noted to be generally lower as compared to completion of treatment itself (Table 1). Studies utilising a comparison group and reporting data completeness between groups described similar completeness levels across these (Depestele et al., 2017; Whitney et al., 2012). Mehl et al. (2013) reported 100% data completion though of note this study had a small sample size (15), used two outcome measures, and data were collected pre‐ and post‐intervention with no further follow‐up. Studies measuring outcomes only pre‐ and post‐interventions reported outcome completion rates in the range of 50%–94% at the post‐intervention time‐point (Depestele et al., 2017; Gelin et al., 2015; Hollesen et al., 2013; Salaminiou et al., 2017; Skarbø and Balmbra, 2020; Stewart et al., 2021). Studies collecting follow‐up data reported completion rates of 31%–81% at this timepoint (Dimitropoulos et al., 2015; Eisler et al., 2016b; Marzola et al., 2015; Whitney et al., 2012; Wierenga et al., 2018).

## 4 DISCUSSION

This review and meta‐analysis investigated the impact of MFT on the mental and physical health of ED patients and their families. Meta‐analyses were conducted separately for studies comparing MFT to another intervention, and studies that adopted a pre/post design. Our meta‐analysis demonstrated MFT resulted in significant benefits in weight gain, ED symptoms, patients' and parents' self‐reported depression symptoms, and reductions in parents' negative experiences of caregiving. Subgroup meta‐analyses comparing adult to adolescent populations found that whilst adolescents showed significant improvements in weight, no significant improvements were found exclusively in the adult samples. This was consistent with our review findings and those described in Baudinet et al. (2021) which also showed MFT reduces ED symptoms as reported by both the patients and parents. However, meta‐analysis found significant improvements were only evident when making comparisons before and after the intervention: MFT did not result in significant improvements when compared to another intervention, most commonly single‐family therapy.

Meta‐analysis revealed no significant improvements in family functioning and positive aspects of caregiving as reported by family members. As initially noted by Baudinet et al. (2021) the review found reductions in the levels of expressed emotion demonstrated in the family, though again MFT did not result in significant improvements compared to control interventions. The review also found there were significant reductions in patient's anxiety though no improvements were seen in parents' ratings of patients' anxiety nor parents' own anxiety. Through its generation of novel data on the size and significance of effects, this meta‐analysis supports the conclusions of a recently published systematic scoping review of qualitative and quantitative evidence‐base for MFT for EDs that MFT is associated with a range of physical and psychological improvements amongst individuals and their families (Baudinet et al., 2021).

Intervention completion rated ranged from 86% to 100%, indicating a high level of acceptability. Questionnaire completion rates were lower suggesting these may not be as tolerable for families to complete as the MFT intervention itself. Taken as a whole, papers looking at the effects of MFT on adolescents versus young adults and amongst inpatient versus outpatient settings report similar findings: trends towards improvements in weight, ED symptoms, and carer burden, though the significance of these trends varied. It must be noted 13 studies assessed MFT as an adjunct intervention: only one 5‐day multi‐family intervention was given in isolation and one study did not specify if it was adjunct to another intervention. Therefore, the unique contributions of MFT cannot be ascertained.

### 4.1 Strengths and limitations

This is the first meta‐analysis to specifically focus on the impact of MFT on adult and adolescent patients with a range of ED diagnoses and their families. Rigorous evaluation of the efficacy of MFT is essential given its global implementation into services (Gelin et al., 2018) and inclusion in NICE guidelines. One previous systematic review of 30 articles reporting adjunct approaches to family‐based treatments focussed on numerous interventions including MFT as well as partial hospital programmes, CBT, DBT, and parent‐focussed sessions. The authors found early evidence augmentative FBT approaches were effective in assisting weight and symptom improvements for adolescents with restrictive EDs but the grouping together of approaches did not allow the authors to recommend specific interventions (Richards et al., 2018). It is also noted how all included studies reported significant outcome results raising concerns of potential publication bias. An additional strength of this review is its inclusion of papers researching adolescents and adults. We are starting to learn that adults are just as likely to develop EDs as adolescents (Davies et al., 2021) meaning it is imperative that we understand the efficacy and acceptability of an intervention across the lifespan.

There are several important limitations which must be acknowledged. First, only articles published in English peer‐reviewed journals were included: this review and meta‐analysis may have been enhanced by the inclusion of unpublished data. The Western‐centric nature of the review, with all data from North America and Europe does not allow for any analysis into cultural comparisons or for implications to be applied globally. Second, there was wide‐ranging variation in the MFT model utilised, the length and structure of interventions, follow‐up periods, and outcome measures used to assess efficacy. In addition, methodological variability prevented pooling all studies together in the meta‐analysis and there was wide variation in the intervention length and number of contact hours. It is possible a dose‐response effect is seen where contact time confounds the efficacy of an intervention (Robinson et al., 2020) therefore contact hours with professionals should be considered in future sub‐group analyses if a sufficient number of trials are available. Third, study quality was generally weak to moderate, with the only two randomised controlled studies receiving a strong global rating using the EPHPP (Eisler et al., 2016b; Whitney et al., 2012). Many of the studies did not utilise a comparison group meaning assessors and researchers could not be blinded when assessing outcomes. Equally, the evaluation of MFT often in one clinic introduced selection bias.

### 4.2 Implications for research

This review highlights key areas for future research. Many of the studies included evaluated data gathered as part of clinical practice meaning the majority lacked a control group and utilised small, potentially unrepresentative samples from one clinic area. To rectify this, future RCTs assessing the effectiveness of MFT compared to the current first line treatments (single‐family therapy for adolescents and cognitive behavioural therapy for adults; National Institute for Health and Care Excellence, 2017) need to be conducted. Whilst most MFT models are designed as adjunct treatments, the active ingredients of MFT contributing to change in patients with EDs and their family members must be better understood. In addition, future research should aim to recruit more diverse samples that are more representative of the population as a whole, with particular regard to ethnicity and family structures.

### 4.3 Implications for practice

This review found some support for the beneficial role of MFT for patients of a range of ages and their families. Family therapy in either a single‐family or multi‐family setting is noted in the NICE guidelines (National Institute for Health and Care Excellence, 2017) for children and adolescents with AN, but not specifically for other EDs nor for adults. Family therapy is not routinely offered in adult services; however, this initial evidence suggests this may be useful, specifically for younger adults still living in the family home. This may be of particular importance in the current climate in the COVID‐19 pandemic resulting in changes in family living situations, and potentially a larger number of young adults living at or working from the family home. Additionally, with the increase of ED diagnoses since the COVID‐19 pandemic (Taquet et al., 2021), approaches which can either enhance or be offered as alternatives too current interventions could be considered. Whilst MFT is a resource‐intensive intervention requiring multiple healthcare professionals, initial data demonstrate it a cost‐effective alternative to inpatient care (Gelin et al., 2018) which has implications for the training and supervision of staff, and resource allocation of services, particularly important for resource‐limited services such as the UK's NHS. The conduction of sufficiently powered trials allowing for sub‐group analyses would enable us to further understand factors associated with gaining the most benefit from MFT.

## CONFLICTS OF INTEREST

The above authors have no conflicts of interest to declare.

## Supporting information

Supplementary Material

## Data Availability

The data that support the findings of this study are available from the corresponding author upon reasonable request.
