# Supplementary material for: Multi‐family therapy for eating disorders across the lifespan: A systematic review and meta‐analysis
Source: Eur Eat Disord Rev. 2022 Jun 21;30(6):723–45. doi: 10.1002/erv.2919 (PMC9796154; doi:10.1002/erv.2919)
Supplement: Supplementary file 1 — Supplementary Material [file ERV-30-723-s001.docx]

Appendices & Supplementary Material

Appendix 1. Search strategy

| Concept | Number | Search Terms |
| --- | --- | --- |
| Concept 1: Population/participants | 1 | feeding and eating disorders |
|  | 2 | eating disorder* |
|  | 3 | anorexi* |
|  | 4 | bulimi* |
|  | 5 | OSFED or other specified feeding or eating disorder |
|  | 6 | EDNOS or eating disorder not otherwise specified |
|  | 7 | ARFID or avoidant restrictive food intake disorder or selective eating disorder |
|  | 8 | BED |
|  | 9 | binge N3 eat* |
|  | 10 | disordered N2 eat* |
|  | 11 | eating N3 difficult* |
|  | 12 | OR/1-11 |
|  |  |  |
| Concept 2: Intervention | 13 | multi family therapy |
|  | 14 | multi-family group therapy |
|  | 15 | multi?family N3 therapy |
|  | 16 | multi-fam* |
|  | 17 | OR/13-16 |
|  |  |  |
| Concept 3: Design | 18 | experimental study or experimental research or quasi experimental study |
|  | 19 | randomi#ed controlled trials or randomi#ed controlled trial or randomi#ed control trial or randomi#ed clinical trial |
|  | 20 | RCT or RCTs |
|  | 21 | random* |
|  | 22 | experiment* |
|  | 23 | clinical N3 trial |
|  | 24 | control* |
|  | 25 | pilot |
|  | 26 | feasibility |
|  | 27 | comparative |
|  | 28 | prospective |
|  | 29 | OR/18-28 |
|  |  |  |
| All concepts combined | 30 | 12 AND 17 AND 29 |

Supplementary Material

Table S1.

*Risk of bias for all studies using the EPHPP tool.*

| Reference | Selection bias | Study design | Confounders | Blinding | Data collection | Withdrawals and drop-outs | Global rating |
| --- | --- | --- | --- | --- | --- | --- | --- |
| Denhagg et al., 2021 | Weak | Moderate | Strong | Weak | Strong | Weak | Weak |
| Depestele et al., 2017 | Moderate | Strong | Strong | Moderate | Strong | Weak | Moderate |
| Dimitropoulos et al., 2015 | Moderate | Strong | Strong | Moderate | Strong | Weak | Moderate |
| Eisler et al., 2016 | Strong | Strong | Strong | Moderate | Strong | Moderate | Strong |
| Gabel et al., 2014 | Moderate | Moderate | Strong | Weak | Strong | N/A | Moderate |
| Gelin et al., 2015 | Moderate | Moderate | Strong | Weak | Strong | Moderate | Moderate |
| Hollesen et al., 2013 | Moderate | Moderate | Strong | Weak | Strong | Moderate | Moderate |
| Marzola et al., 2015 | Strong | Moderate | Weak | Weak | Strong | Strong | Weak |
| Mehl et al., 2013 | Moderate | Moderate | Strong | Weak | Weak | Strong | Weak |
| Salaminiou et al., 2017 | Strong | Moderate | Strong | Weak | Strong | Strong | Moderate |
| Skarbø & Balmbra, 2020 | Weak | Moderate | Strong | Weak | Weak | Strong | Weak |
| Stewart et al., 2021 | Moderate | Moderate | Strong | Weak | Strong | Weak | Weak |
| Tantillo et al., 2019 | Moderate | Moderate | Strong | Weak | Strong | Strong | Moderate |
| Whitney et al., 2012 | Moderate | Strong | Strong | Moderate | Strong | Moderate | Strong |
| Wierenga et al., 2018 | Strong | Moderate | Strong | Weak | Strong | Strong | Moderate |

Table S2.

*Risk of bias in RCTs using the RoB 2 tool*

| Reference | Bias arising from randomisation process | Bias due to deviations from intended interventions | Bias due to missing outcome data | Bias in measurement of the outcome | Bias in selection of the reported result | Overall risk of bias rating |
| --- | --- | --- | --- | --- | --- | --- |
| Eisler et al., 2016 | Low | Low | Low | Low | Some concerns | Some concerns |
| Whitney et al., 2012 | Some concerns | Low | Low | Low | Some concerns | Some concerns |


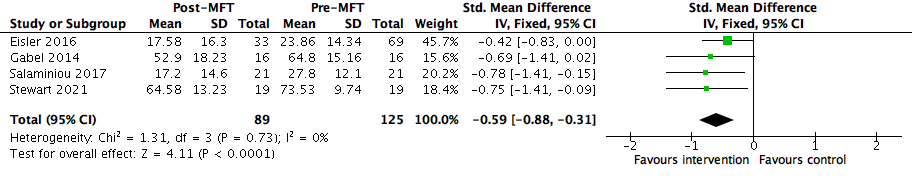


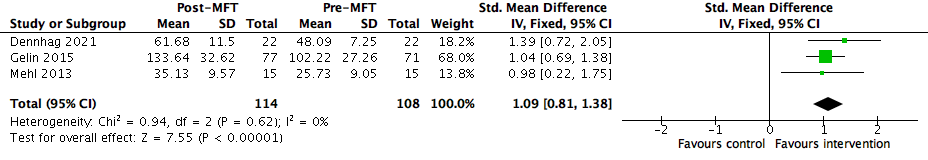


*Figure S1.* Table and forest plot of pooled data comparing the change in self-reported depression symptoms (top) and psychological well-being after receiving MFT (bottom).


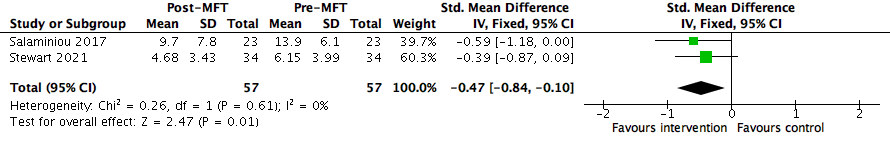


*Figure S2.* Table and forest plot of pooled data comparing the change in family members’ mood and well-being after receiving MFT.

*
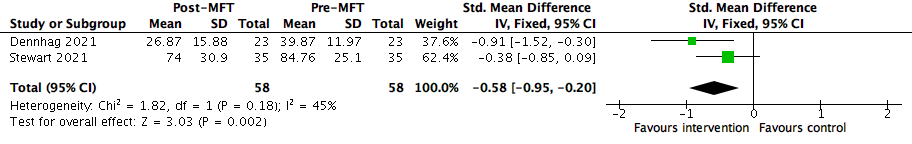
*

*Figure S3.* Table and forest plot of pooled data comparing the change in parent’s reported negative appraisals of caregiving depression symptoms after receiving MFT.
